# Implantation of Transvenous Permanent Pacemaker in a Patient with Persistent Left Superior Vena Cava and Absent Right Superior Vena Cava

**Published:** 2013-12-01

**Authors:** Mohammad Javad Alemzadeh-Ansari, Akbar Shafiee, Ahmad Yaminisharif

**Affiliations:** 1 Cardiology Department, Tehran Heart Center, Tehran University of Medical Sciences, Tehran, IR Iran

**Keywords:** Superior Vena Cava, Vascular Malformations, Central Venous Catheterization, Pacemaker

## Abstract

Vascular access has remained a major challenge for implantation of permanent pacemaker leads. A persistent Left Superior Vena Cava (LSVC), especially with an absent Right Superior Vena Cava (RSVC), is a rare finding during pacemaker implantation and is accompanied by technical difficulties. Herein, we describe a case of sinoatrial node arrest, in which finding a suitable vein for passing the lead was challenging. The patient had a persistent LSVC with an absent RSVC.

## 1. Introduction

Vascular access still continues to be a major challenging issue in implantation of permanent pacemaker leads, particularly in the patients with vascular variations. A persistent Left Superior Vena Cava (LSVC) is a very rare venous malformation which can create trouble during pacing lead implantation (1, 2). We report a case of sinoatrial node arrest, in which finding a suitable vein for passing the lead was challenging. The patient had a persistent LSVC with an absent Right Superior Vena Cava (RSVC).

## 2. Case Report

A 58 year-old man was admitted to our hospital complaining about dizziness and lightheadedness for seven days. He had no history of chest pain or loss of consciousness. He had undergone coronary artery angiography three months ago following a myocardial infarction, which revealed significant stenosis (90%) at proximal portion of the left anterior descending artery, while the other coronary arteries were normal. The Left Ventricular (LV) ejection fraction was 35% in left ventriculography with severe hypokinesia of the anterior wall. In that admission, the patient was treated with percutaneous coronary intervention and a Cypher stent (3 × 35 mm) was implanted in the proximal portion of the left anterior descending artery. Until seven days before the new admission, the patient was symptom free.

**Figure 1.**
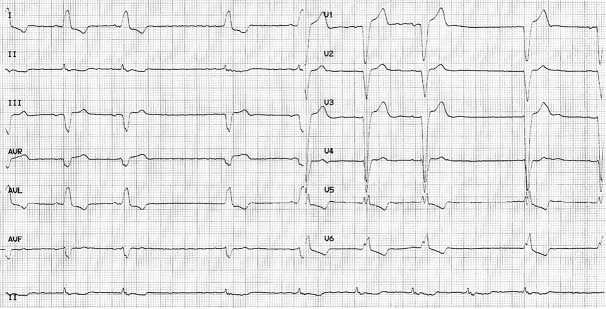
The Surface 1-Leads Electrocardiogram Revealed Sinus Pause with Junctional Escape Beat. Morphology of QRS Wave Was Left Bundle Branch Block.

The blood pressure at presentation was 120 / 50 mmHg, while the pulse rate was 40 beats per minute. The surface 12-lead Electrocardiogram (ECG) revealed a sinus P wave rate of 60 / min, but a sinus pause up to 3000 milliseconds (ms) with junctional escape beat was also observed (Figure 1). The morphology of QRS wave was left bundle branch block. After stopping the use of digoxin and carvedilol for 72 hours, a 24-hour Holter ECG monitoring was requested which revealed frequent episodes of sinus pauses of up to 3500 ms. In the pre-implantation echocardiography, LV ejection fraction was 40%. Therefore, we decided to implant a dual-chamber permanent pacemaker (DDDR mode) for the patient.

### 2.1. Implantation Procedure

**Figure 2.**
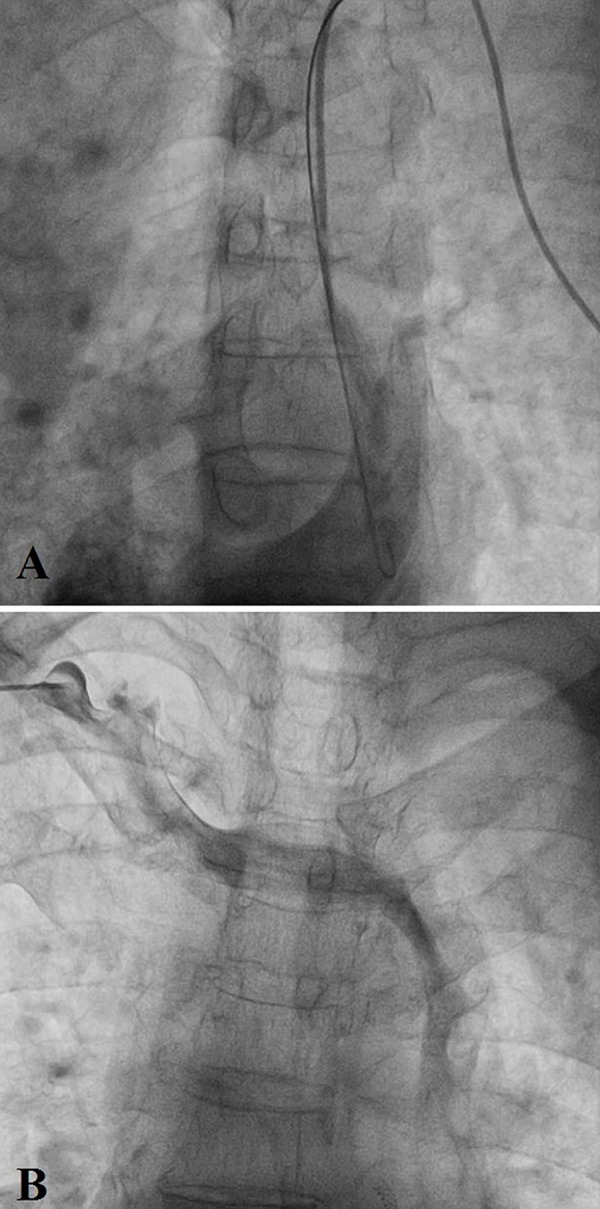
Persistence Left Superior Vena Cava and Absence of Right Superior Vena Cava. A) The Venography from Left Subclavian Vein Revealed the Presence of Persistence Left Superior Vena Cava. B) The Venography from Right Subclavian Vein: Dye Passed Through Innominate Vein and Then Drained into Persistence Left Superior Vena Cava.

**Figure 3.**
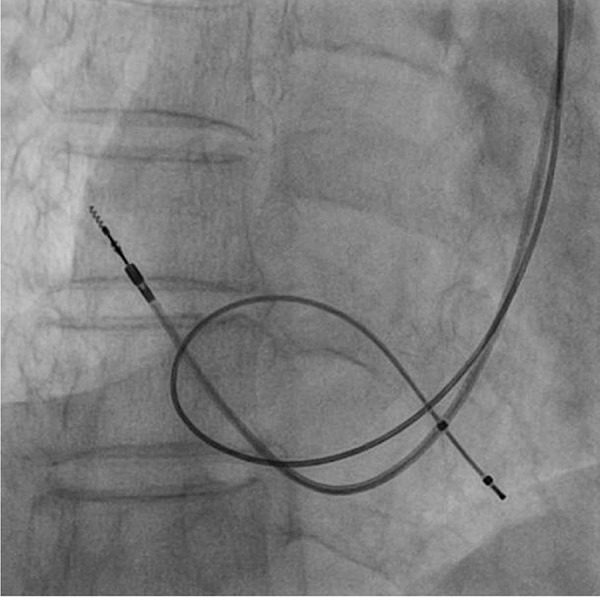
The Right Atrium and Right Ventricle Leads Implantation Was Performed via the Persistent Left Superior Vena Cava.

The approach via the left subclavian vein for the implantation of the pacemaker leads resulted in an unexpected entrance into the right atrium through the coronary sinus vein. Venography from the left subclavian vein revealed that the dye drained into the right atrium through an LSVC and the coronary sinus vein (Figure 2A). Consequently, we decided to implant the pacemaker leads through the right subclavian vein. Interestingly, the dye drained into the LSVC via the innominate vein in the venography of the right subclavian vein and revealed the absence of the RSVC (Figure 2B). At this point, we decided to approach via the left subclavian for the lead implantation. A 58-cm Medtronic 5076 CapSureFix Novus lead (Medtronic, Inc., Minneapolis, MN, USA) was placed in the right atrium and an LV pacing lead with an over-the-wire technique (EASYTRAK model 4518, Guidant Corp, St. Paul, Minnesota) was positioned in the right ventricle using the loop technique (Figure 3). Attempts to implant the LV lead in one of the coronary sinus branches were not successful. The leads were thereafter connected to the VEDR01 Versa DR generator (Medtronic, Minneapolis, USA) in the DDDR mode. The patient was discharged in a good condition and the 1-year follow-up was eventless. The pacemaker was working properly with acceptable pacing and sensing in both atrial and ventricular leads (V- pacing = 0.5 V, pulse width = 0.46 ms).

## 3. Discussion

A persistent LSVC is estimated to be present in 0.3 - 0.5% of the general population and in 5 - 10% of the patients with congenital heart defects (1, 3). Development of a persistent LSVC is a complex embryological process and results from the persistence of the embryonic left anterior cardinal vein (1). During the fourth week of gestation, the venous blood of the head and the upper half of the body are drained by bilateral symmetrically arranged veins: the left and right anterior cardinal veins. At the eighth week of gestation, the left brachiocephalic vein develops as a bridge between the two cardinal veins. The portion of the left anterior cardinal vein below this anastomosis usually obliterates and degenerates, leaving only the right anterior cardinal vein which becomes anastomosed to the superior vena cava (3, 4). Absence of the RSVC is exceedingly rare with this anomaly, as was the case in our patient, and is reported in approximately less than 0.1% of the general population (5).

A precise knowledge of a persistent LSVC is virtually important, particularly when central catheterization via the subclavian or internal jugular vein is difficult. In case of suspicion, transthoracic two-dimensional echocardiography can be employed as a diagnostic tool for this extremely rare congenital anomaly. The coronary sinus vein is dilated and opacified before the right side of the heart following bolus injections of contrast material from a left arm vein (6, 7).

For diagnosing this anomaly, imaging modalities, such as chest radiograph, venography, computerized tomography, and magnetic resonance imaging, can be utilized. On the plain chest radiograph, the catheter can be detected running down the left mediastinal border. Also, widening of the aortic shadow, a paramediastinal bulge, a paramediastinal strip, or crescent along the upper left cardiac border can be discovered (8). A contrast-enhanced computerized Tomography can confirm the diagnosis by enhancement of the coronary sinus (3). In our case, we diagnosed this abnormality following venography. The venography revealed that the left subclavian vein drained into the persistent LSVC and then into the coronary sinus. Furthermore, the right subclavian vein drained into the persistent LSVC via the innominate vein.

In this particular case, we had to use an LV lead instead of regular RV leads as LV leads are longer and this could help us to perform the lead implantation. Also, active LV lead was not available at the time of implantation. Of course, we are aware that use of an active lead could prevent lead dislodgement in future.

Persistent LSVC in the absence of the RSVC complicates pacemaker lead implantation via the transvenous approach, rendering the procedure more technically difficult because of the acute angle between the coronary sinus ostium and the tricuspid valve and causing lead instability through the left cephalic vein and the subclavian vein approach (9-12). Also, traversing the tricuspid valve and obtaining a stable position in the right ventricle usually requires prolonged manipulation.

A persistent LSVC with an absent RSVC is a rare congenital abnormality that can complicate the insertion of the permanent pacemaker lead. We showed that placement of the leads via the left subclavian vein allowed stable dual-chamber pacing.
